# Plasma Membrane Targeting of Endogenous NKCC2 in COS7 Cells Bypasses Functional Golgi Cisternae and Complex N-Glycosylation

**DOI:** 10.3389/fcell.2016.00150

**Published:** 2017-01-04

**Authors:** Richa Singh, Shams Kursan, Mohamed Y. Almiahoub, Mohammed M. Almutairi, Tomás Garzón-Muvdi, Francisco J. Alvarez-Leefmans, Mauricio Di Fulvio

**Affiliations:** Department of Pharmacology and Toxicology, Boonshoft School of Medicine, Wright State UniversityDayton, OH, USA

**Keywords:** NKCC2A, Golgi, N-glycosylation, cell volume, COS7

## Abstract

Na^+^K^+^2Cl^−^ co-transporters (NKCCs) effect the electroneutral movement of Na^+^-K^+^ and 2Cl^−^ ions across the plasma membrane of vertebrate cells. There are two known NKCC isoforms, NKCC1 (*Slc12a2*) and NKCC2 (*Slc12a1*). NKCC1 is a ubiquitously expressed transporter involved in cell volume regulation, Cl^−^ homeostasis and epithelial salt secretion, whereas NKCC2 is abundantly expressed in kidney epithelial cells of the thick ascending loop of Henle, where it plays key roles in NaCl reabsorption and electrolyte homeostasis. Although NKCC1 and NKCC2 co-transport the same ions with identical stoichiometry, NKCC1 actively co-transports water whereas NKCC2 does not. There is growing evidence showing that NKCC2 is expressed outside the kidney, but its function in extra-renal tissues remains unknown. The present study shows molecular and functional evidence of endogenous NKCC2 expression in COS7 cells, a widely used mammalian cell model. Endogenous NKCC2 is primarily found in recycling endosomes, Golgi cisternae, Golgi-derived vesicles, and to a lesser extent in the endoplasmic reticulum. Unlike NKCC1, NKCC2 is minimally hybrid/complex N-glycosylated under basal conditions and yet it is trafficked to the plasma membrane region of hyper-osmotically challenged cells through mechanisms that require minimal complex N-glycosylation or functional Golgi cisternae. Control COS7 cells exposed to slightly hyperosmotic (~6.7%) solutions for 16 h were not shrunken, suggesting that either one or both NKCC1 and NKCC2 may participate in cell volume recovery. However, NKCC2 targeted to the plasma membrane region or transient over-expression of NKCC2 failed to rescue NKCC1 in COS7 cells where NKCC1 had been silenced. Further, COS7 cells in which NKCC1, but not NKCC2, was silenced exhibited reduced cell size compared to control cells. Altogether, these results suggest that NKCC2 does not participate in cell volume recovery and therefore, NKCC1 and NKCC2 are functionally different Na^+^K^+^2Cl^−^ co-transporters.

## Introduction

The Na^+^K^+^2Cl^−^ co-transporters (NKCCs) are membrane proteins that carry out the electroneutral movement of Na^+^, K^+^, and 2Cl^−^ ions across the plasma membrane. These symporters belong to the *Slc12a* family of solute carriers, which comprise at least eight homologous genes: *Slc12a1* (NKCC2), *Slc12a2* (NKCC1), *Slc12a3* (Na^+^-Cl^−^ co-transporter, NCC), *Slc12a4-7* (K^+^-Cl^−^ co-transporters, KCC1-4), and *Slc12a9* (Hartmann et al., 2014). NKCCs and NCCs actively accumulate Cl^−^ in cells, using the energy stored in the sum of the Na^+^, K^+^, and Cl^−^ chemical potential gradients, whereas KCCs mediate active Cl^−^ extrusion driven by the sum of the K^+^ and Cl^−^ chemical potential gradients. NKCCs and KCCs play important roles in cell volume regulation, in the maintenance of the intracellular chloride concentration ([Cl^−^]_i_) and in transepithelial solute and water transport (Alvarez-Leefmans, 2012).

NKCC1 is a ubiquitously distributed protein (Gamba, 2005). Molecular and/or functional expression of NKCC1 and its spliced variants has been demonstrated in a variety of primary cells types, including cell lines (Alshahrani et al., 2012; Mao et al., 2012; Markadieu and Delpire, 2014; Singh et al., 2015). In contrast, the main products of the *Slc12a1* gene (NKCC2) i.e., NKCC2A, NKCC2B, and NKCC2F, have long been considered to be exclusive to the apical membrane of the tubular cells of the thick ascending loop of Henle (TALH). In this location, NKCC2 plays a key role in salt reabsorption and urine concentration (Castrop and Schiessl, 2014). Mutations in the human *SLC12A1* gene underlie neonatal Bartter's syndrome type I, a disorder characterized by severe dehydration, polyuria and electrolyte imbalance (Simon et al., 1996). Although there is no doubt that NKCC2 is abundantly expressed in the kidney and in cell lines derived from the TALH (Eng et al., 2007) or the macula densa (Fraser et al., 2007), there is growing evidence showing relatively low levels of expression of extra-renal NKCC2. For instance, NKCC2 expression has been reported in enteric neurons (Xue et al., 2009), gastric, intestinal, endolymphatic sac, and olfactory epithelia (Akiyama et al., 2007, 2010; Nickell et al., 2007; Nishimura et al., 2009; Xue et al., 2009; Zhu et al., 2011; Ji et al., 2012), starburst amacrine cells (Gavrikov et al., 2006), chondrocytes (Bush et al., 2010), and endocrine/neuroendocrine cells including insulin secreting β-cells of the pancreas (Corless et al., 2006; Bensellam et al., 2009; Ghanaat-Pour and Sjöholm, 2009; Alshahrani et al., 2012; Alshahrani and Di Fulvio, 2012) and vasopressinergic/oxytocinergic neurons of the supraoptic and paraventricular nuclei (Hindmarch et al., 2006; Konopacka et al., 2015).

Little is known about the functional role of extra-renal NKCC2. In some cell types, NKCC2 is co-expressed with NKCC1, but whether these proteins interact remains to be determined. NKCC2 expression, plasma membrane localization and function all increase in vasopressinergic and oxytocinergic neurons expressing NKCC1 in rats subjected to chronic dehydration (Hindmarch et al., 2006; Konopacka et al., 2015). These data suggest that the *Slc12a1* gene is responsive to osmotic stress. In line with the latter, absence of NKCC1 in β-cells results in permanent cell shrinkage and increased insulin secretion by mechanisms related to increased NKCC2 expression (Alshahrani and Di Fulvio, 2012; Alshahrani et al., 2015). NKCC1 and NKCC2 are not functionally equivalent; although both proteins transport the same ions with the same stoichiometry (Gamba et al., 2009), NKCC1 actively co-transports ~550 molecules of water per cycle (Hamann et al., 2010), whereas NKCC2 is a “dry” co-transporter; it does not transport water (Zeuthen and Macaulay, 2012).

Although the molecular determinants of these functional differences between NKCC1 and NKCC2 are unknown, we recently observed that knocking down NKCC1 in COS7 cells resulted in increased NKCC2 expression that correlated with NKCC2 immunolabeling near or at the plasma membrane (Alshahrani et al., 2015). Since targeting of endogenous NKCC1 to the plasma membrane is independent of hybrid/complex N-glycosylation (Singh et al., 2015) and genetic deletion of NKCC1 in some cells results in permanent cell shrinkage (Crum et al., 2012), we hypothesized that NKCC2 expression increases in cells subjected to sustained osmotic shrinkage by mechanisms that do not require the classic secretory pathway. In the present report, we confirm and extend previous results by demonstrating that: (i) one splice variant of NKCC2, NKCC2A, is produced in COS7, (ii) NKCC2 is natively expressed in COS7 cells at relatively low levels, (iii) NKCC2 and NKCC1 co-localize to some but not all cellular compartments, (iv) under basal conditions endogenous NKCC2 localizes to the endoplasmic reticulum (ER), cis/medial Golgi cisternae, pericentriolar/microtubule organizing center, and endosomal compartments but not to lysosomes or the plasma membrane region, (v) NKCC2 expression and plasma membrane region localization increase in response to sustained exposure to hyperosmotic solutions and (vi) complex N-glycosylation or functional Golgi stacks are not required for NKCC2 targeting to the plasma membrane region. Altogether, these data shed new light on the cellular and molecular mechanisms involved in membrane trafficking and osmotic sensitivity of extra-renal NKCC2.

## Materials and methods

### Materials

*Pfx* DNA polymerase, RNase-OUT, SuperScript-III reverse transcriptase, random hexamers, Lipofectamine2000 transfection reagent, and baculovirus-mediated transduction systems for Golgi detection (Bacman II) were from Invitrogen (Carlsbad, CA); dNTPs, alkaline phosphatase and exonuclease I were from Affimetrix/USB (Cleveland, OH); custom primers were from Integrated DNA Technologies (Coralville, IA); the RNeasy minikit was from Qiagen (Valencia, CA). General chemicals were from Sigma (Saint Louis, MO). Tissue culture media, serum and supplements were from Thermo-Fisher (Waltham, MA).

### Antibodies

Monoclonal antibodies against tubulin (6G7) and lysosomal-associated membrane protein-1 (LAMP, 2G9) were from Developmental Studies Hybridoma Bank (DSHB, University of Iowa). Rabbit anti-human β-actin antibodies were from Cell Signaling Technology (Danvers, MA). Purified mouse anti-Rab11 was from BD Transduction Labs (San Jose, CA). Anti-calreticulin (CRT) and chicken anti-human NKCC1 antibodies were from Thermo-Scientific (Rockford, IL). Anti-NKCC2 antibodies were kindly provided by Dr. Pablo Ortiz (Henry Ford Hospital, Detroit MI). Specific antibodies against activated NKCC2 i.e., phospho-residue S^126^ were kindly provided by Dr. Mark Knepper (Laboratory of Kidney and Electrolyte Metabolism, NIH). Monoclonal antibodies against α-mannosidase II (ManII) were a kind gift of Dr. Brian Burke (Institute of Medical Biology, Singapore). Rabbit polyclonal anti-FLAG antibodies were from GenScript (Piscataway, NJ). The FITC-conjugated *Maackia amurensis* lectin (MAL) was from Vector Labs (Burlingame, CA). Conjugated secondary antibodies for immunofluorescence microscopy or Western blotting were from Jackson Immunoresearch (West Grove, PA).

### Cell culture, transfection, and stable silencing

NKCC1-expressing COS7 cells (ATCC, Manassas, VA) were grown and maintained in 6-well plates (BioLite, Thermo Scientific), in DMEM supplemented with 10% FBS and antibiotics. Cells were cultured in 5% CO_2_-95% air at 37°C. The medium was changed every 2–3 days until confluence. Plasmid transfection into COS7 cells was performed using Lipofectamine2000 following the manufacturer's instructions. Full-length human NKCC2A (hNKCC2A) cDNA cloned from human dorsal root ganglion cells (*GenBank* accession number EF559316) was used to create expression plasmids. The cloning plasmids harboring hNKCC2A cDNA wild type (hNKCC2A^WT^) or mutants i.e., non-glycosylatable (hNKCC2A^N445/455Q^) or lacking the last 181 residues (hNKCC2A^ΔC^), were developed in the laboratory of one of us (FJA-L). These cDNAs were introduced into pcDNA3.1 mammalian expression vectors and FLAG-tagged. Lentiviral vectors expressing mCherry-tagged hNKCC2A^WT^ were from GeneCopoeia (Rockville, MD). To silence endogenous NKCC1 or NKCC2 expression, COS7 cells were stably transfected with human lentiviral constructs encoding green fluorescent protein (GFP) and shRNAs against the 4th exon of hNKCC1, as previously described (Alshahrani et al., 2015; Singh et al., 2015) or against the 20th exon of hNKCC2. As control, we used constructs lacking shRNA sequences.

### RNA extraction and RT-PCR

First-strand cDNA synthesis from total RNA and gene-specific PCR was done as described in detail elsewhere (Alshahrani et al., 2012; Grobe et al., 2015; Singh et al., 2015). Control RT-PCR reactions were performed using glyceraldehyde phosphate dehydrogenase (GAPDH, NM_002046) primers: GAPDH-555 sense: GTG AAG GTC GGA GTC AAC GGA TTT, GAPDH-555 antisense: CAC AGT CTT CTG GGT GGC AGT GAT. The PCR primers used to amplify NKCC2 as overlapping fragments of the whole open reading frame (ORF, nucleotide position: 1 to 3300) and partial un-translated sequences (5′- and 3′-UTRs, positions–218 to 1 and 3301 to 3819, respectively) of NKCC2A are shown in Supplementary Table 1. Primer design and *in silico* analysis of sequences obtained were performed using Geneious R9 (Biomatters Ltd., Auckland, New Zealand).

### Western blotting

Fifty to one hundred micrograms of total protein from COS7 cells were subjected to SDS-PAGE (pre-casted gels 4–20% Pierce, Thermo Scientific, Rockford, IL), transferred to PDVF membranes and blotted as described in detail elsewhere (Singh et al., 2015). β-actin expression was used as internal loading control. Western blots were developed by the ECL method (Supersignal West Pico, Pierce) using HRP-conjugated secondary antibodies.

### Immunofluorescence and cell cross sectional area measurements

COS7 cells were grown on glass coverslips placed on 6-well plates and immunolabeled as previously described (Singh et al., 2015). Immunolabeled cells attached to coverslips were mounted using Vectashield containing 4′-6-diamidino-2-phenylindole (DAPI, Vector Labs, Burlingame, CA) to counterstain the cell nuclei. Slides were visualized in an Olympus Epi Fluorescence microscope with RT color camera using 60x or 100x oil objectives and appropriate fluorescence filters or an FV1000 Confocal Microscope (Olympus, PA, USA). Non-confocal micrographs were obtained using a digital camera (Diagnostics Instrument Spot 6 digital). Cell edges were visualized on digital pictures (RGB, 600dpi) using *ImageJ* software (NIH) and applying the Canny-Deriche filtering plugin. Co-localization was estimated by applying the co-localization colormap plugin of *ImageJ* to 8-bit transformed images. Cell surface area (squared pixels) was obtained using the free-hand selection tool to draw cell edges and transformed to μm^2^ based on a microscope scale set at 600x magnification (1350 pixels = 125 μm).

### Bumetanide-sensitive Cl^−^ uptake

The total intracellular content of Cl^−^ was determined in non-confluent COS7 cells grown in 12 well-plastic plates using calibrated ion-selective electrodes (Orion-Thermo Scientific, Rockford, IL), as described previously (Singh et al., 2015). Briefly, cells were washed and depleted of endogenous Cl^−^ at room temperature in isotonic (ISO, ~300 mOsm/Kg H_2_O) media of the following composition (in mM): 0.83 Na_2_HPO_4_, 1 Mg_2_SO_4_, 20 HEPES, 10 mannose, 130, 5 and 2 Na^+^, K^+^, and Ca^2+^ gluconate salts, respectively. After incubation for 1 h in this Cl^−^-free media, cells were exposed to control medium containing physiological Cl^−^ concentrations i.e., gluconate salts were replaced with equimolar amounts of NaCl, KCl, and CaCl_2_, and cells were allowed to accumulate Cl^−^ ions for 5 min. The uptake process was terminated placing cells on ice and washing them in ice-cold Cl^−^-free ISO solutions. The total cellular Cl^−^ content was determined potentiometrically in neutralized 0.25 N NaOH cellular extracts, calculated and expressed as nmol of Cl^−^ per μg of total protein. The bumetanide (BTD)-sensitive Cl^−^ uptake was defined as the difference between Cl^−^ accumulated under control conditions to that obtained in the presence of 10 μM BTD.

### Statistical analysis

Analysis of multiple group differences was performed by one-way analysis of variance (ANOVA) followed by Student-Newman-Keuls' test. *P*-values lower than 0.05 were considered of statistical significance.

## Results

### COS7 cells express a single splice variant of NKCC2

**Figure 1:**
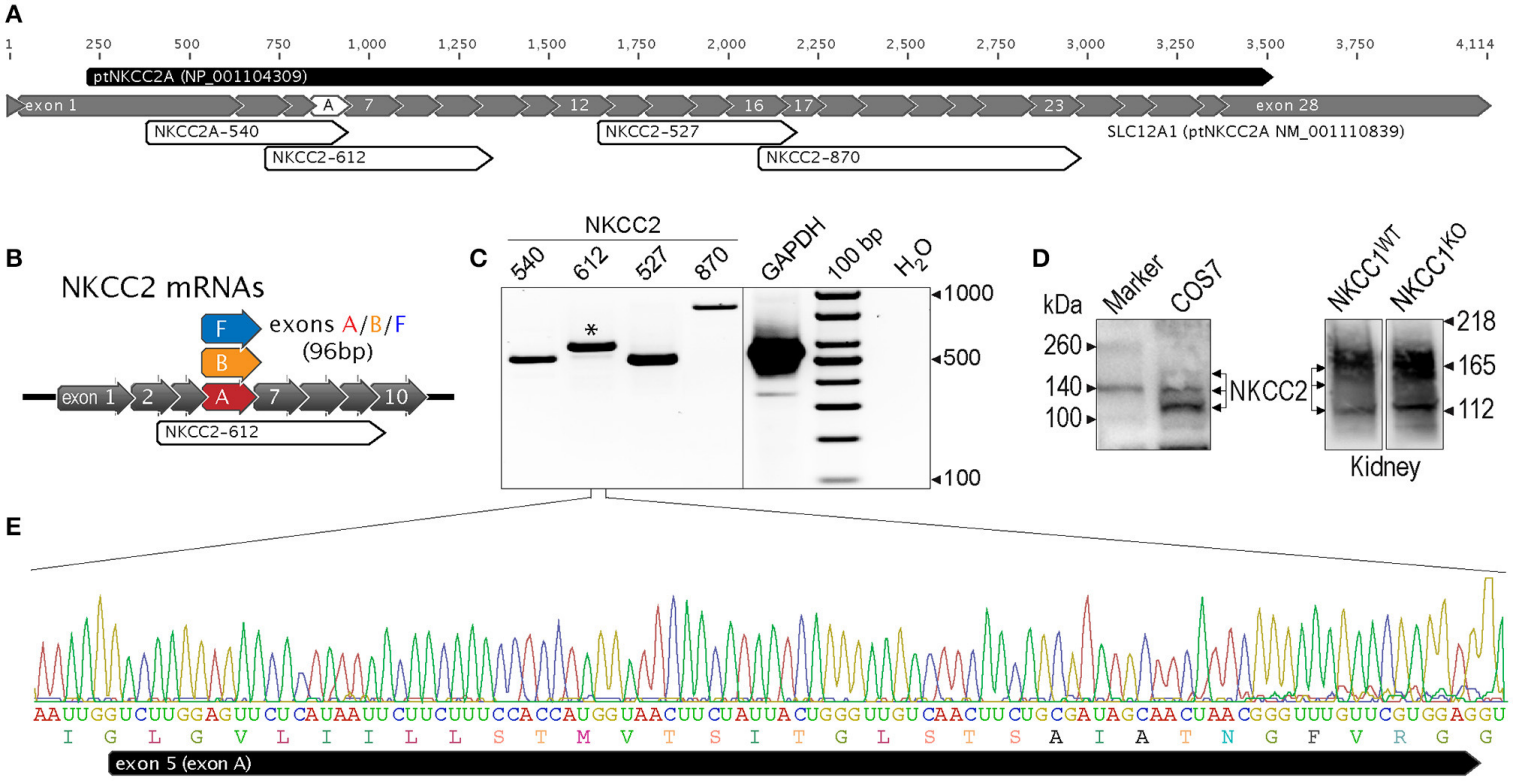
**Endogenous NKCC2A expression in COS7 cells. (A)** Representation of chimpanzee NKCC2A mRNA sequence of reference (ptNKCC2A, *RefSeq* NM_001110839), inferred from genomic sequences. The 28 coding exons are represented as gray filled arrows, numbered relative to the first coding exon (*exon 1*). The protein predicted *in silico* has 1099 amino acids and it is represented as a black-filled arrow on top of this panel. The predicted RT-PCR fragments amplified using the NKCC2 primer sets are represented as white arrows, and are labeled as follows: NKCC2A-540, NKCC2-612, NKCC2-527, and NKCC2-870. **(B)** Representation of mouse NKCC2 splicing variants mNKCC2A, mNKCC2B, and mNKCC2F showing the encompassing primer set NKCC2-612, predicted to co-amplify NKCC2A, B and F. **(C)** Representative RT-PCR experiment performed using NKCC2 primer sets indicated in *B*. Shown is a 2% agarose gel showing bands of predicted size for NKCC2 i.e., 540, 612, 527, and 870 bps. RT-PCR reactions included amplification of GAPDH (555 bp). Negative controls were performed using H_2_O instead of RNA and GAPDH-555 primer set. **(D)** Immunoblot of COS7 lysates (*left*) showing NKCC2 protein expression as bands of ~120kDa, ~135kDa, and ~165kDa. As positive control for NKCC2 expression, whole mouse kidney lysates obtained from WT and NKCC1^KO^ mice (*right panel*) were run in parallel. **(E)** Partial nucleotide sequence of the RT-PCR amplicon obtained with NKCC2-612 primer set. The band of 612 bp (*asterisk*) was sequenced in both directions. The sequence chromatogram shown is representative of 6 different amplification reactions and corresponds to the region encompassing exon A of COS7 NKCC2A mRNA.

Using immunolabeling methods it was shown that COS7 express low but significant levels of NKCC2 (Alshahrani et al., 2015). However, the splice variant/s of NKCC2 were not identified. In the present paper we used RT-PCR and specific primer sets (Supplementary Table 1) designed to amplify known splice variants of the co-transporter i.e., NKCC2A, NKCC2B, and NKCC2F. Bands of predicted sizes were amplified from total RNA obtained from COS7 cells demonstrating expression of NKCC2 transcripts. Figure 1E shows the partial nucleotide sequence of the NKCC2-612 fragment predicted to co-amplify NKCC2A, B and F transcripts (Figure 1B). *In silico* analysis of the entire sequence of this RT-PCR fragment demonstrated 98% identity to hNKCC2A or chimpanzee's NKCC2A (ptNKCC2A). Thus, the NKCC2 variant endogenously expressed in COS7 cells is likely NKCC2A. Exon B or F sequences were not detected in this 612 bp fragment suggesting that neither NKCC2B nor NKCC2F are expressed in COS7 cells. Further, the nucleotide sequences of RT-PCR fragments of 540 bp confirmed the presence of NKCC2A (*not shown*). To provide additional evidence of NKCC2A expression in COS7 cells, the complete open-reading frame (ORF) plus 3'/5'-UTR sequences corresponding to NKCC2A mRNAs were PCR-amplified in overlapping fragments and fully sequenced in both directions. The predicted translation product and topology of the nucleotide sequence of NKCC2A expressed in COS7 (*GenBank* accession number: JQ708180) is shown in Supplementary Figure 1. *In silico* analysis and comparison of NKCC2 cDNAs cloned from COS7 with the human NKCC2A sequence of reference (NM_000338) and with hNKCC2A cloned from dorsal root ganglion cells (EF559316) or kidney (U58130) reveals >98% pairwise identity between cDNA and ORF sequences (*not shown*).

To extend these results at the protein level, NKCC2 was immuno-detected in blots of protein extracts from COS7 cells and kidney tissues from WT mouse and mouse lacking NKCC1 (NKCC1^WT^ and NKCC1^KO^, respectively) as controls for potential cross-contamination with NKCC1. As shown in Figure 1D, endogenous NKCC2 is detected mainly as a band of ~120 kDa corresponding to core/high mannose version of the transporter. Additional bands of ~140–170 kDa were also detected. The latter bands correspond to hybrid/complex N-glycosylated NKCC2. Immunoblots from kidney proteins confirmed the well-known band pattern of NKCC2 i.e., two bands, one of ~120 kDa and the other of ~165 kDa (Paredes et al., 2006). Taken together, these results confirm and extend previous data (Alshahrani et al., 2015) and suggest that the NKCC2 expressed in COS7 cells is NKCC2A and that most of it corresponds to the core/high mannose N-glycan version of the transporter.

### Endogenous NKCC2 is not localized to the plasma membrane region of COS7 cells

**Figure 2:**
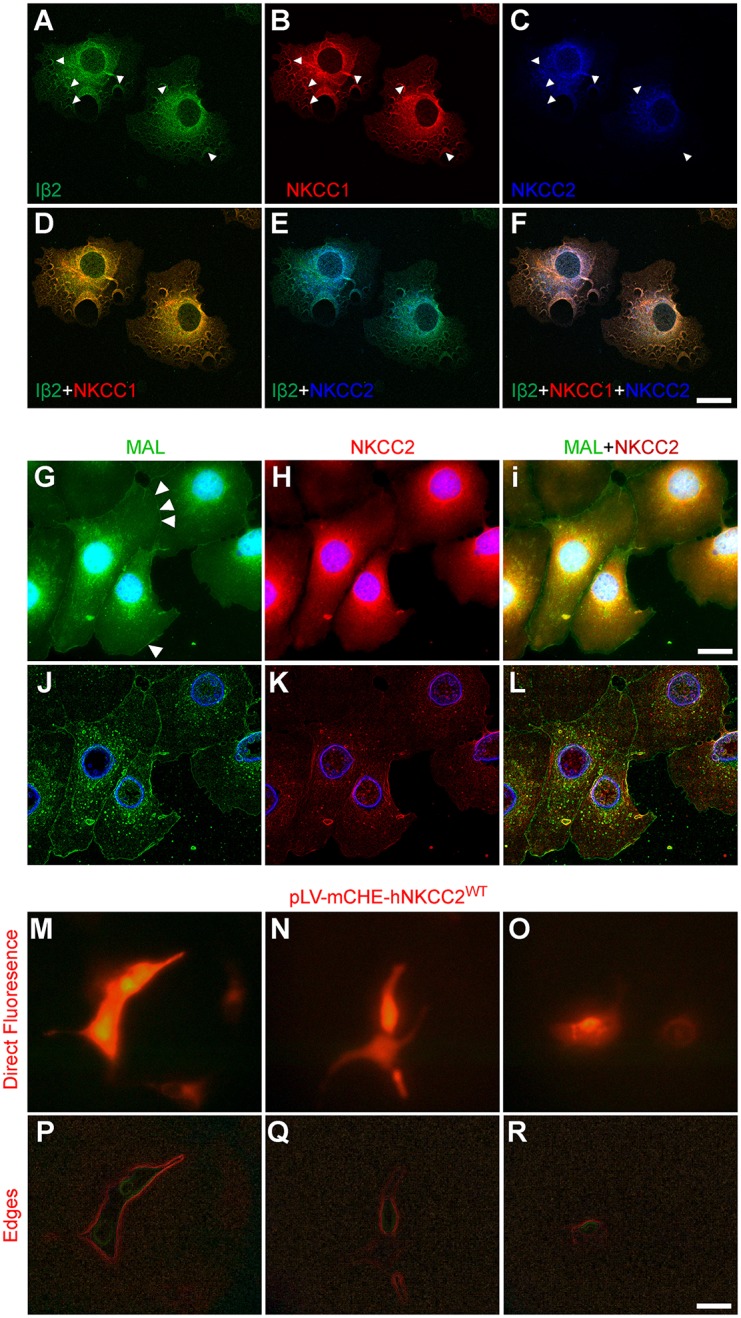
**Endogenous NKCC2 does not localize to the plasma membrane region of COS7 cells under basal normotonic conditions. (A–C)** Confocal immunofluorescence microscopy images of sub-confluent COS7 cells immunolabeled with primary antibodies directed against integrin β2 (Iβ2, **A**), NKCC1 **(B)**, and NKCC2 **(C)**. The secondary antibodies were conjugated to AF488 (green), Cy3 (red), and DyLight405 (blue), respectively. **(D)** Overlays of (**A**; Iβ2) and **(B**; NKCC1). **(E)** Overlays of (**A**; Iβ2) and **(C)**. **(F)** Overlays of (**A;** Iβ2), **(B**; NKCC1), and **(C**; (NKCC2). **(G–I**) Immunofluorescence microscopy images of fixed and permeabilized COS7 cells labeled with FITC-conjugated *Maackia Amurensis* lectin (MAL, **G**) to visualize cell membranes (arrowheads). Cells were also probed against NKCC2 coupled to Cy3-conjugated secondary antibodies **(H)** and the images superimposed **(I)**. In (**G–I**), cell nuclei were counterstained using DAPI. **(J–L)** Visualization of cell edges of images **(G–I)** by using *ImageJ* and applying the Canny-Deriche filtering plugin. Note the lack of NKCC2-IR in the overlay image shown in (**L)**. **(M–O)** Direct immunofluorescence microscopy of COS7 cells growing in glass coverslips and over-expressing different levels of the lentiviral vector mCherry-NKCC2A^WT^. **(P,R)** Visualization of the edges of the transfected cells shown in **(M–O)** by applying the Canny-Deriche filtering plugin of *ImageJ*. All images were taken at 600x magnification. Bar represents 10 μm.

NKCC2 is found widespread in intracellular compartments of non-epithelial cells including hypothalamic neurons (Konopacka et al., 2015), insulin-secreting β-cells (Alshahrani et al., 2012; Alshahrani and Di Fulvio, 2012), or COS7 cells in culture (Alshahrani et al., 2015). To identify the intracellular compartments in which NKCC2 is expressed in COS7, triple immunolabeling of NKCC2, integrin β2 (Iβ2), and NKCC1 was performed. Iβ2 is a putative plasma membrane marker whereas NKCC1 is not only expressed in the plasma membrane but also in the endoplasmic reticulum (ER), Golgi cisternae and endosomes of COS7 cells (Singh et al., 2015). As expected, both Iβ2-IR (Figure 2A) and NKCC1-IR (Figure 2B) were found in or in close proximity to the plasma membrane as well as in perinuclear compartments. As shown in Figure 2D, Iβ2- and NKCC1-IR extensively overlapped. However, NKCC2-IR (Figure 2C) does not exhibit an expression pattern consistent with plasma membrane localization in these cells. In fact, NKCC2 is mainly found in Iβ2- or NKCC1-negative compartments (Figure 2F) and it is barely detected in the plasma membrane region of COS7 cells. To obtain further evidence for the lack of endogenous NKCC2 expression in the plasma membrane, NKCC2 was co-immunolabeled with fluorescein-conjugated *M. amurensis* lectin (MAL), a carbohydrate binding protein used to label plasma membrane-associated carbohydrate moieties (Wu et al., 2006). The results show that NKCC2 and MAL do not co-localize further suggesting that plasma membrane localization of the co-transporter is minimal or undetectable under basal control conditions in COS7 cells (Figures 2G–L). To determine if this immunolocalization pattern correlates with the relatively low expression levels of endogenous NKCC2, COS7 cells grown in glass coverslips were transfected with a lentiviral vector encoding mCherry-tagged hNKCC2A^WT^ (~4 μg). Cells exhibiting different levels of expression of mCherry-tagged hNKCC2A^WT^ were imaged using direct immunofluorescence microscopy (Figures 2J–L) and digital images were filtered to detect cells' edges. Figures 2M–R show that over-expressed hNKCC2A^WT^ localizing toward the cell's edges increases with the expression levels of hNKCC2A^WT^. This observation suggests that plasma membrane localization of NKCC2 is a function of its expression levels.

### Endogenous NKCC2 localizes in intracellular vesicular compartments

**Figure 3:**
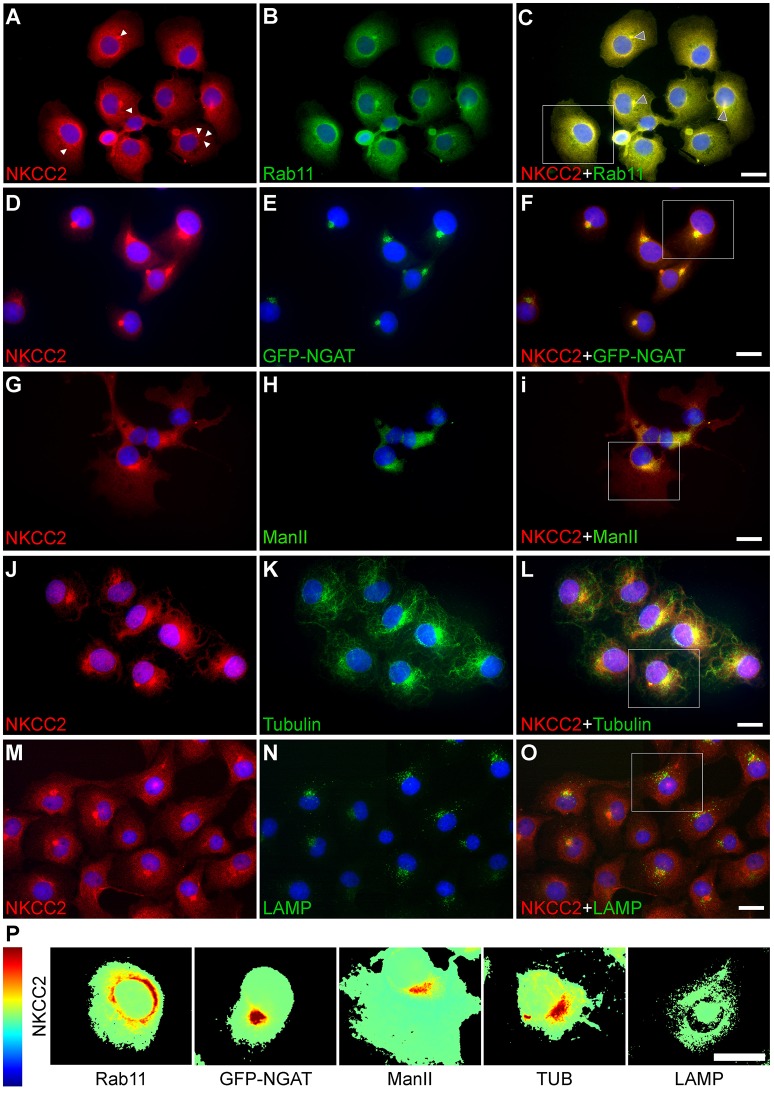
**Endogenous NKCC2 localizes to recycling endosomes, Golgi cisternae, and tubulin-positive structures**. Immunofluorescence microscopy images of sub-confluent COS7 cells grown in glass coverslips were permeabilized and immunolabeled with markers of intracellular organelles. **(A–C)** COS7 cells labeled with primary antibodies against NKCC2 **(A)** and Rab11, a marker of the recycling endosomal compartment **(B)**. Co-localization of NKCC2 and Rab11 is shown in the merged image **(C)**, where the withe arrowheads indicate NKCC2 localization in structures lying at one of the poles of the cell or in vesicles. **(D–F)** COS7 cells transduced with GFP-NGAT constructs to visualize the Golgi compartments **(E)** and then permeabilized and immunolabeled against NKCC2 **(D, F)**. **(G–I)** COS7 cells immunolabeled against NKCC2 **(G)** and α-mannosidase II, a marker of trans-Golgi cisternae (ManII, **H**). Localization of NKCC2 to Golgi compartments is shown in the respective merged images (**F** and **I)**. **(J–L)** COS7 cells immunolabeled against NKCC2 **(J)** and tubulin **(K)** showing localization of the transporter to structures resembling the MTOC/pericentriolar compartment **(L)**. **(M–O)** Expression of NKCC2 **(M)** and the lysosomal marker LAMP **(N)** were detected but not co-localized **(O)**. The cell nuclei were counterstained with DAPI in all micrographs. All micrographs were taken at 600x. Bar represents 10 μm. **(P)** Co-localization heat-map of the cells framed with a white rectangle in **(C**, **F**, **I**, **L**, and **O)**, computed by using the NIH *ImageJ* co-localization plugin.

To identify the intracellular compartments where NKCC2 is localized, double immunolabeling experiments were performed using Rab11, a marker of perinuclear recycling endosomes and Golgi-derived vesicles (Ullrich et al., 1996). As shown in Figures 3A–C, endogenous NKCC2 extensively co-localize in Rab11-positive vesicles. However, NKCC2-IR was also observed in intracellular compartments other than Rab11-positive vesicles. These compartments appeared as discrete structures near one of the nuclear poles and in small cytoplasmic vesicles (Figure 3A, white arrows). These compartments may represent the microtubule organizing center (MTOC)/pericentriolar compartment (PC), Golgi stacks or lysosomes (Lippincott-Schwartz, 1998). To define Golgi localization, endogenous NKCC2 was immunolabeled in COS7 cells either transduced with GFP-tagged N-acetyl-galactosaminyl transferase-2 (GFP-NGAT), a *cis*-Golgi resident enzyme, or co-immunolabeled with α-mannosidase II (ManII), a *trans*-Golgi resident enzyme (Burke et al., 1982). Figures 3D–I show NKCC2A co-localization with GFP-NGAT and ManII, demonstrating that the co-transporter locates in Golgi cisternae. Furthermore, as shown in Figures 3J–L, endogenous NKCC2 partially co-localized with tubulin [TUB, microtubule marker (Desai and Mitchison, 1997)] at one of the poles of the cell but not in radiating tubulin-positive structures or in compartments positive for the lysosomal marker LAMP (Figures 3M–O). The extent of NKCC2 co-localization with Rab11, GFP-NGAT, ManII, TUB, or LAMP is shown in their respective heat-maps (Figure 3P). Taken together, these results strongly suggest that endogenous NKCC2 in COS7 cells is mainly expressed as an intracellular protein localized in endosomal vesicular compartments, Golgi cisternae and in structures resembling the MTOC/PC.

### Endogenous NKCC2 trafficking does not require functional golgi compartments in COS7 cells

**Figure 4:**
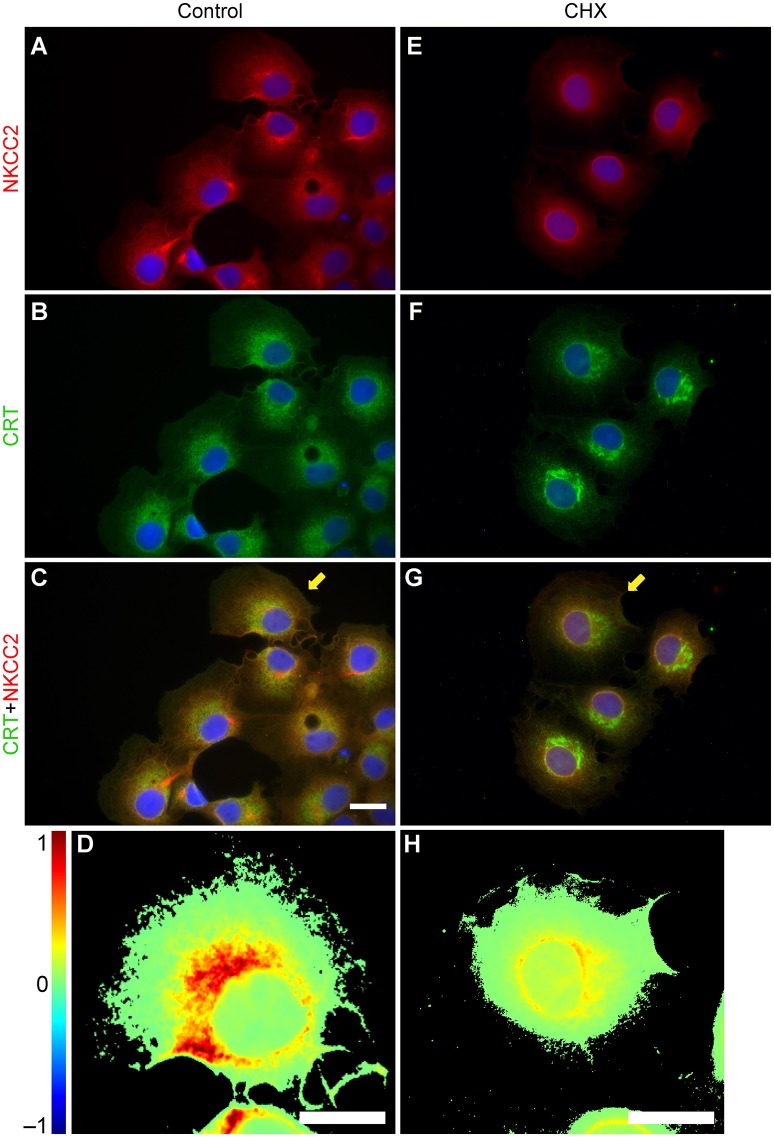
**NKCC2 localizes to the ER of COS7 cells. (A–D)** COS7 cells immunolabeled against NKCC2 **(A)** or calreticulin (CRT, **B**), a calcium-binding protein that resides in the ER. Secondary antibodies were: Cy3 (NKCC2, red) and AF488 (CRT, green). The co-localization of NKCC2 and CRT is shown in **C**, a merged image of **(A,B)**, and in the heat-map shown in **(D)**, which corresponds to the cell labeled with a yellow arrow. **(E–H)** Images of COS7 cells grown in the presence of cycloheximide (CHX, 1 μg/ml, 2 h) to acutely inhibit ongoing protein synthesis in the ER. NKCC2 **(E)** and CRT **(F)** were detected by using the relevant primary antibodies and secondary antibodies labeled with Cy3 (red) and AF488 (green). Merged images **(E,F)** show degree of co-localization on heat-maps **(G–H)**.

Since most of the NKCC2 in COS7 cells appears as a core/high mannose N-glycosylated protein i.e., ~120 kDa (Figure 1D), the transporter is expected to be localized in ER, where all proteins are synthesized. This hypothesis was tested using double immunolabeling of NKCC2 and calreticulin (CRT), an ER-resident chaperone. As shown in Figure 4, NKCC2 and CRT co-localize in COS7 cells cultured under control basal conditions (Figures 4A–C). In fact, when protein synthesis was inhibited by exposing the cells for 2 h to cycloheximide the expression of NKCC2 in the ER was significantly reduced (Figures 4D–F). This can be more clearly visualized in co-localization heat-maps (Figures 4D–H).

**Figure 5:**
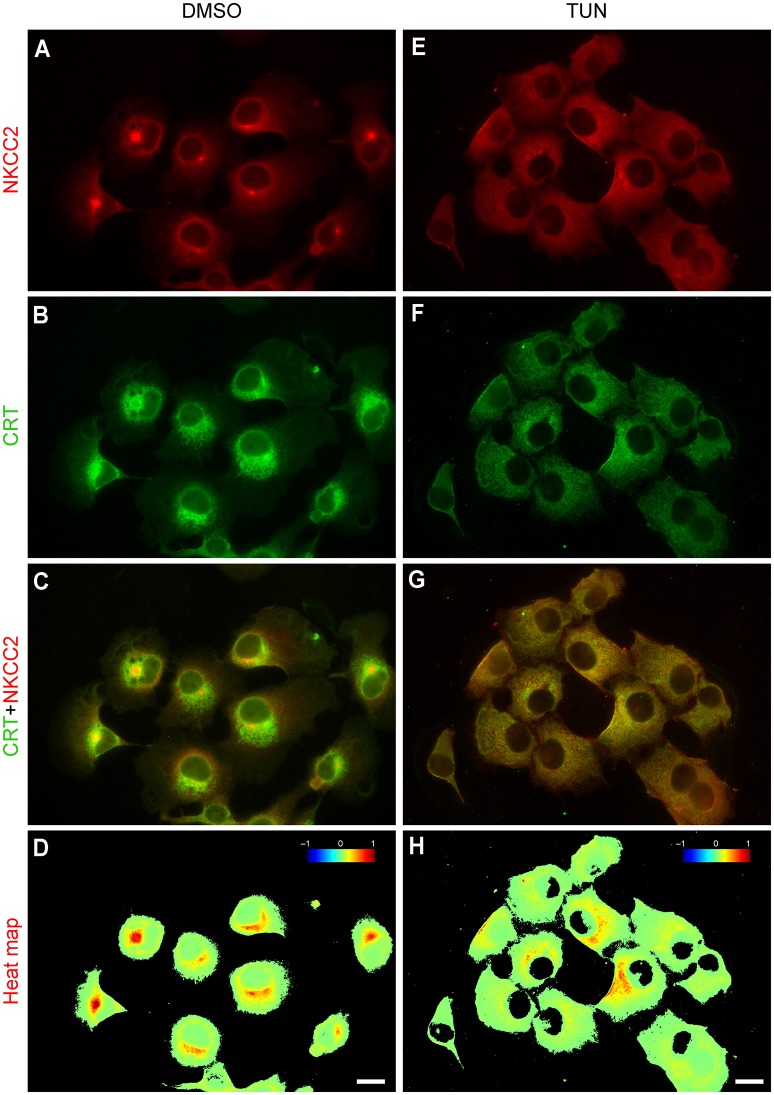
**Inhibition of N-glycosylation results in redistribution of endogenous NKCC2 in COS7 cells**. Immunofluorescence microscopy images of COS7 cells grown under control conditions (DMSO 0.2% used as vehicle, 16 h, **A–D**) or treated with 2 μg/ml tunicamycin (TUN plus vehicle, 16 h **E–H**). NKCC2 and CRT co-localization is shown in merged images **(C,G)**, which were used to compute their respective co-localization heat maps **(D,H)**. Bars represent 10 μm.

**Figure 6:**
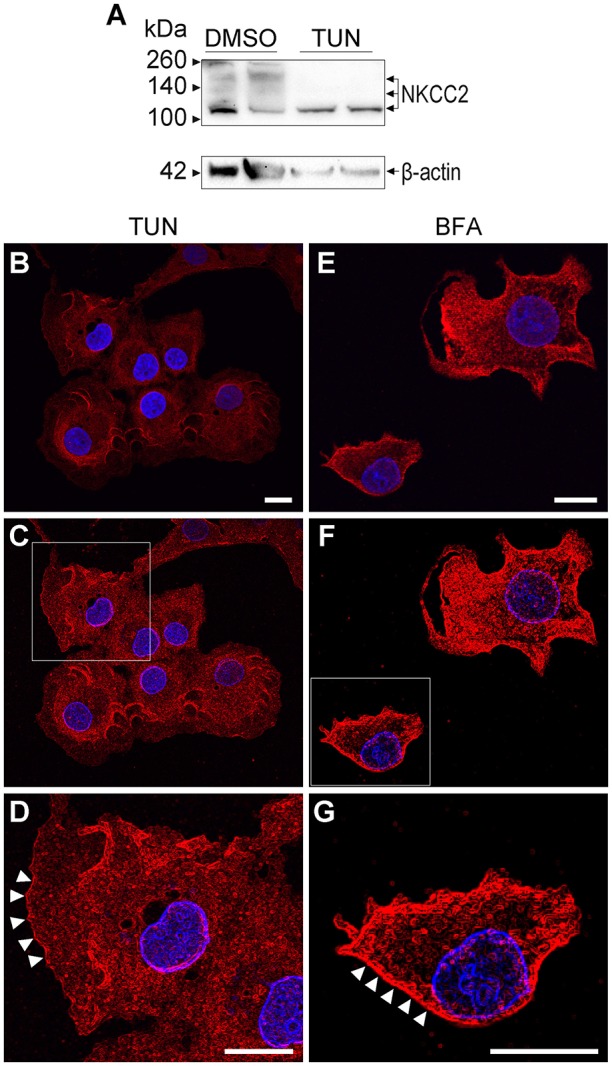
**The first step of N-glycan biosynthesis impairs N-glycosylation of NKCC2 but not its expression or trafficking. (A)** Representative immunoblots showing NKCC2 expression in protein extracts from COS7 cells that were grown under control conditions (0.2% DMSO) or treated with TUN (2 μg/ml, 16 h). Note the cotransporter's bands of ~120 kDa, ~135 kDa, and ~160 kDa in control immunoblots. In contrast, protein extracted from cells treated with TUN only showed a band at ~120 kDa. β-actin was used as loading control. **(B–D)** Confocal images of COS7 cells grown in control conditions (DMSO 0.2% used as vehicle) immunolabeled against NKCC2 and visualized with Cy3-conjugated secondary antibodies (red). Bars in **(B,D)** represent 10 μm. The edges of COS7 cells were visualized by applying the Canny-Deriche filtering plugin of *ImageJ* **(C)** and the cell within the square frame magnified **(D)** to visualize the plasma membrane region, where NKCC2-IR is indicated with arrowheads. **(E–G)** Confocal images of COS7 cells cultured in the presence of brefeldin-A (BFA 1 μg/ml 16 h, **E**) to collapse Golgi membranes into the ER. NKCC2 was visualized using Cy3-conjugated secondary antibodies (red). The edges of COS7 cells were visualized as indicated in **(C)**. The square in **(F)** indicates the cell magnified in **(G)**, where the plasma membrane location of NKCC2 is indicated with arrowheads. Bars in **(E,G)** represent 10 μm.

To determine the impact of the first step in N-glycan biosynthesis on NKCC2 stability and intracellular trafficking, COS7 cells were treated for 16 h with vehicle (0.2% DMSO) or with vehicle plus tunicamycin (TUN, 2 μg/ml), a blocker of the first step of N-glycan biosynthesis. As shown in Figures 5A–C, NKCC2 and CRT co-localized in control DMSO-treated COS7 cells to an extent similar to that shown in Figure 4, indicating that DMSO does not visibly impact endogenous NKCC2 immuno-localization. However, in contrast with what has been shown for NKCC1 in COS7 cells (Singh et al., 2015), treatment with TUN did not result in evident NKCC2 degradation or retention in the ER. Moreover, NKCC2 appeared completely deglycosylated in immunoblots from proteins extracted from TUN-treated COS7 cells (Figure 6A). Immunofluorescence microscopy revealed that in cells treated with TUN, NKCC2 appeared as vesicle-like punctate structures throughout the cytoplasm (Figures 6B,C), and surprisingly, it was also clearly localized in plasma membrane region (Figures 6B–D). These results indicate that endogenous NKCC2 in COS7 cells targeting to the plasma membrane is independent of N-glycans biosynthesis.

To determine whether the co-transporter is able to bypass the classic ER-Golgi secretory route, NKCC2 immunolocalization was studied in COS7 cells treated with brefeldin-A (BFA, 2 μg/ml, 16 h), an antibiotic that inhibits retrograde cargo transport from Golgi to ER and anterograde delivery from post-Golgi compartments (Grieve and Rabouille, 2011). As shown in Figures 6E–G, in COS7 cells treated with BFA NKCC2 appears localized at or near the plasma membrane. These results indicate that NKCC2 may traffic directly from the ER to the plasma membrane.

### NKCC2 expression pattern changes upon sustained osmotic stress

**Figure 7:**
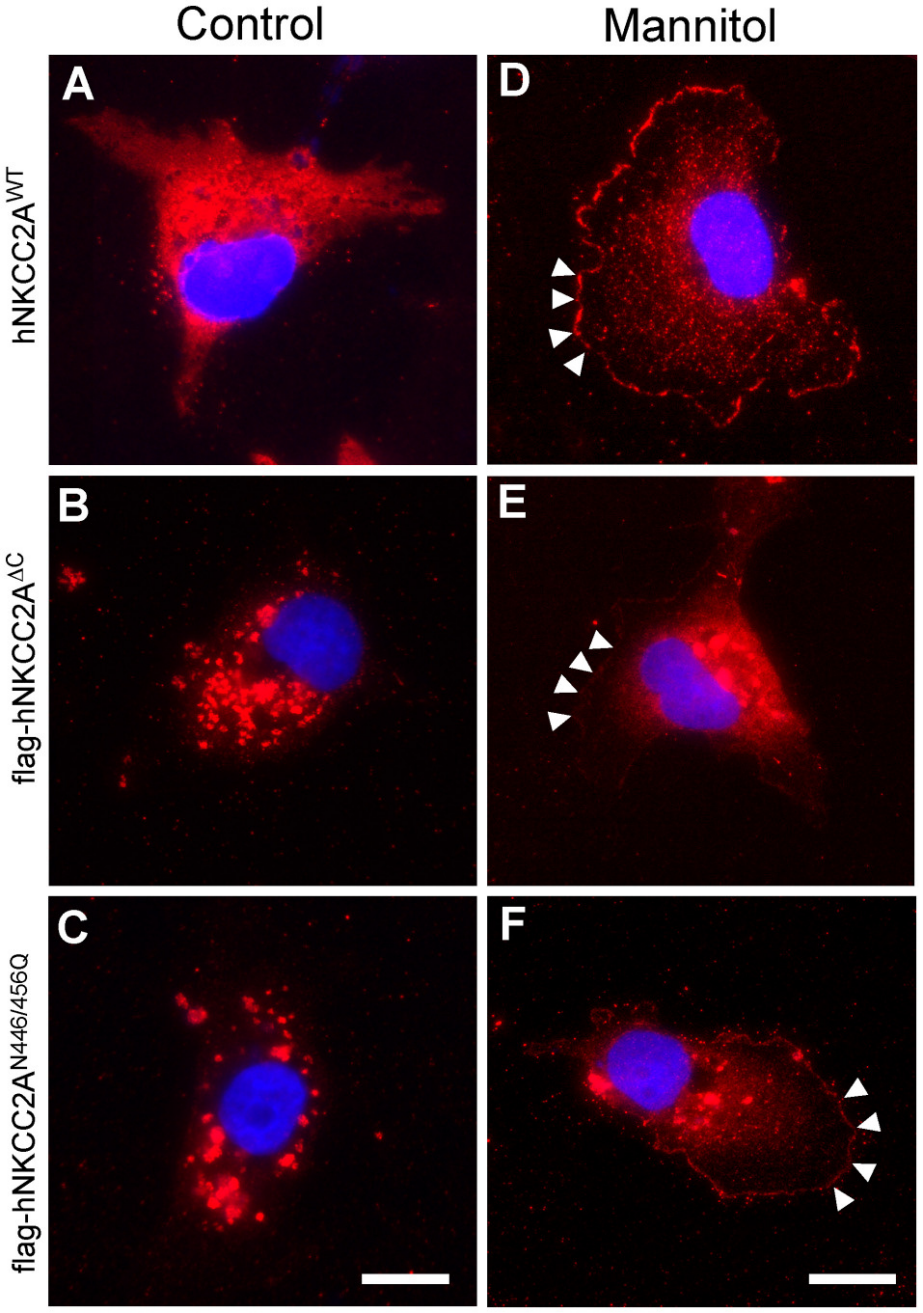
**Sustained hyperosmotic stress promotes NKCC2A plasma membrane localization independently of N-glycosylation**. Immunofluorescence microscopy images of COS7 cells transfected with FLAG-tagged hNKCC2A^WT^ **(A)**, hNKCC2A^ΔC^ **(B)**, or hNKCC2A^N446/456Q^ **(C)** growing on glass coverslips in normotonic conditions (~300 mOsm/kg H_2_O) or in hyperosmotic (~6.7%) media (320 mOsm/kg H_2_O) for 16 h. The hyperosmotic media was prepared by adding 19.5 mM mannitol to control media **(D–F)**. Cells were labeled by using FLAG antibodies coupled to Cy3-conjugated secondary antibodies. Arrowheads in **(D–F)** represent FLAG-NKCC2 expressed toward the edges of the cells. Bar represents 10 μm and applies to **(E,D)**.

Since NKCC2 reaching of the plasma membrane region of COS7 cells is independent of functional Golgi and chronic osmotic stress increase NKCC2 expression in COS7 cells (Alshahrani et al., 2015), here we explored whether NKCC2 lacking N-glycans traffics to the plasma membrane of osmotically stressed cells. To this end, COS7 were transiently transfected with ~2 μg of FLAG-tagged hNKCC2A^WT^, hNKCC2A^ΔC^ [a truncated mutant lacking the last 181 C-terminal residues, known to be retained in the ER when over-expressed in cells (Zaarour et al., 2009)] or hNKCC2A^N446/456Q^ [a non-glycosylatable mutant unable to reach the plasma membrane when over-expressed in mammalian cells (Benziane et al., 2007)]. Forty-eight hours post-transfection, cells were incubated in isotonic serum-free medium (~300 mOsm/Kg H_2_O) alone, or for 16 h in slightly hyperosmotic (~6.7%) serum-free medium prepared by adding 19.5 mM mannitol giving a final osmolality of ~320 mOsm/Kg H_2_O. Cells were then fixed and processed for immunofluorescence microscopy using anti-FLAG antibodies. As shown in Figure 7A, transiently over-expressed hNKCC2A^WT^ localizes in intracellular compartments rather than in or near the plasma membrane, whereas hNKCC2A^ΔC^ (Figure 7B) or hNKCC2A^N446/456Q^ (Figure 7C) were retained in intracellular structures, as previously shown (Benziane et al., 2007; Zaarour et al., 2009). Interestingly, long-lasting (16 h) exposure of the transfected cells to slightly hyperosmotic medium resulted in a redistribution of hNKCC2A^WT^ toward the edges of the cells retargeting intracellularly retained NKCC2A mutants. Altogether, these results suggest that NKCC2A traffics to the plasma membrane in response to long-lasting incubation in slightly hyperosmotic media and that this phenomenon does not require functional Golgi cisternae.

### NKCC2 expression in response to sustained hyperosmotic stress does not recover cell size

**Figure 8:**
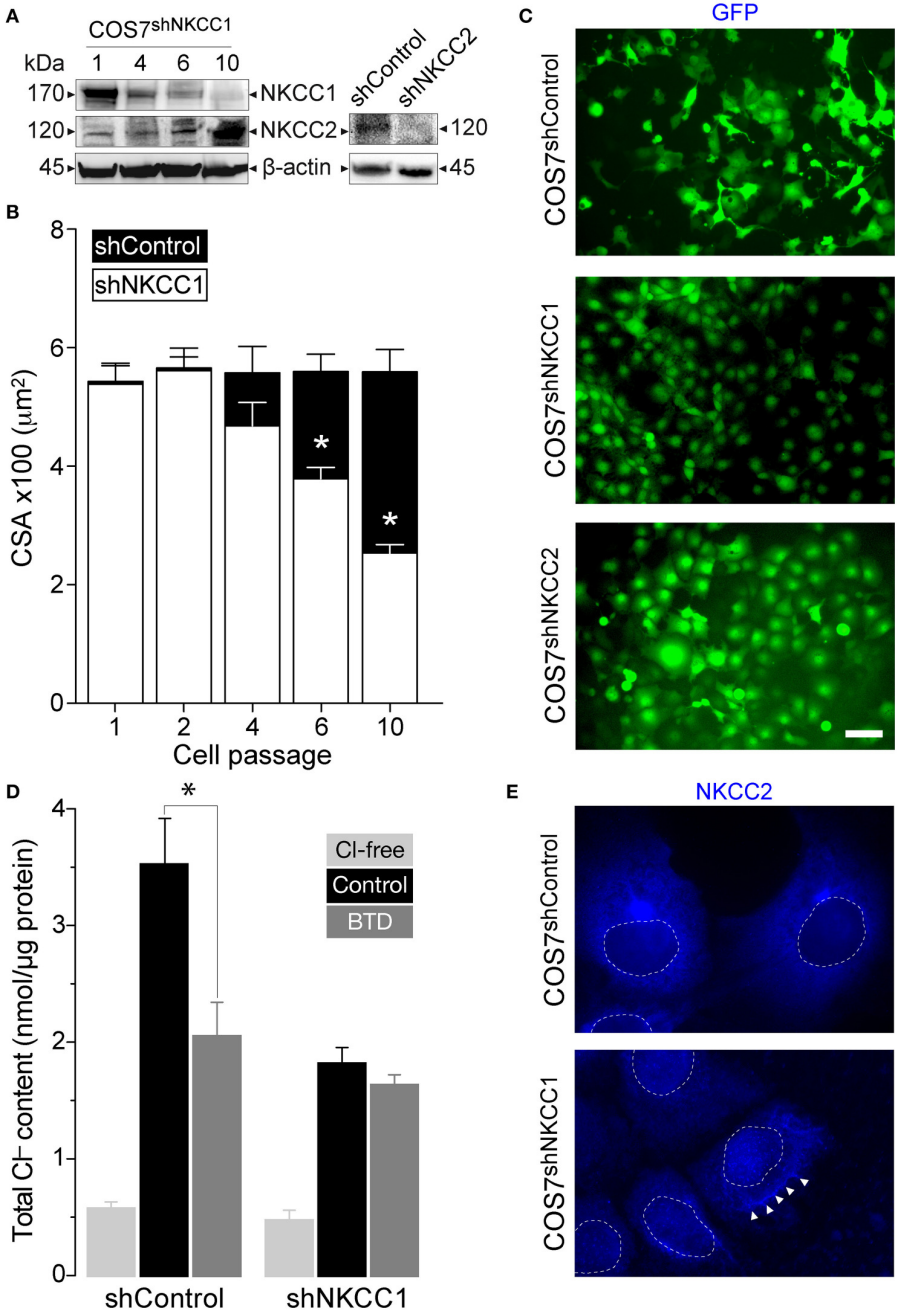
**COS7 in which NKCC1 was silenced were reduced in size. (A)** Immunoblots of protein extracts obtained from COS7^shNKCC1^ cells at indicated post-transfection cell passages. Immunoreactive bands were visualized using anti-NKCC1 and -NKCC2 antibodies coupled to HRP-conjugated secondary antibodies. As controls of NKCC2 expression, protein extracts of COS7^shControl^ and COS7^shNKCC2^ cells were immunoblotted against NKCC2. **(B)** Cell areas of COS7^shControl^ (mock transfected, filled bars) and COS7^shNKCC1^ cells (open bars). Cells at the indicated post-transfection passages (1–10) were plated on coverslips and their cross sectional area (CSA) determined the day after, using *ImageJ*. Asterisks represent statistical significance respect to COS7^shControl^ cells at the same passage number (*p* < 0.05, *n* > 100 cells per group). **(C)** Direct fluorescence microscopy images of confluent COS7^shControl^ (*top*), COS7^shNKCC1^ (*center*), and COS7^shNKCC2^ cells (*bottom*) stably expressing GFP to visualize the reduced overall cell size of COS7^shNKCC1^ cells (p10). Bar represents 50 μm. **(D)** Total and BTD-sensitive components of Cl^−^ uptake into COS7^shControl^ and COS7^shNKCC1^ cells cultured under isotonic conditions in the absence of Cl^−^ (light gray bars), physiological concentrations of Cl^−^ without (black bars) or with 10 μM BTD (dark gray bars). Results are expressed as nmol/μg protein ± SEM (*n* = 5). **(E)** Fluorescence microscopy images of COS7^shControl^ (*top*) and COS7^shNKCC1^ cells (*bottom*) where immunolabeled NKCC2 was detected using Dylight405-conjugated secondary antibodies. Dashed circles denote the cell nuclei. Arrowheads indicate NKCC2 location toward the plasma membrane.

Molecular elimination or pharmacological down-regulation of NKCC1 in COS7 or insulin-secreting β-cells results in increased NKCC2 expression (Alshahrani et al., 2015). Interestingly, these cells are permanently smaller than WT cells, suggesting that NKCC2 expression does not result in cell size recovery. To gain insights into the potential functional role of NKCC2 in NKCC1-silenced COS7 cells [COS7^shNKCC1^, (Singh et al., 2015)], we explored the relation between NKCC2 up-regulation and cell size as a function of shRNA-mediated NKCC1 silencing. As shown in Figure 8A, gradual silencing of NKCC1 in COS7 cells results in parallel up-regulation of NKCC2 expression, whereas the mean cell area of COS7^shNKCC1^ cells is significantly reduced when compared to that of COS7^shControl^. These results can be further observed in the micrographs shown in Figure 8C, where GFP-expressing COS7^shNKCC1^, but not COS7^shNKCC2^ cells, exhibits reduced cell size when compared to that of COS7^shControl^ cells.

**Figure 9:**
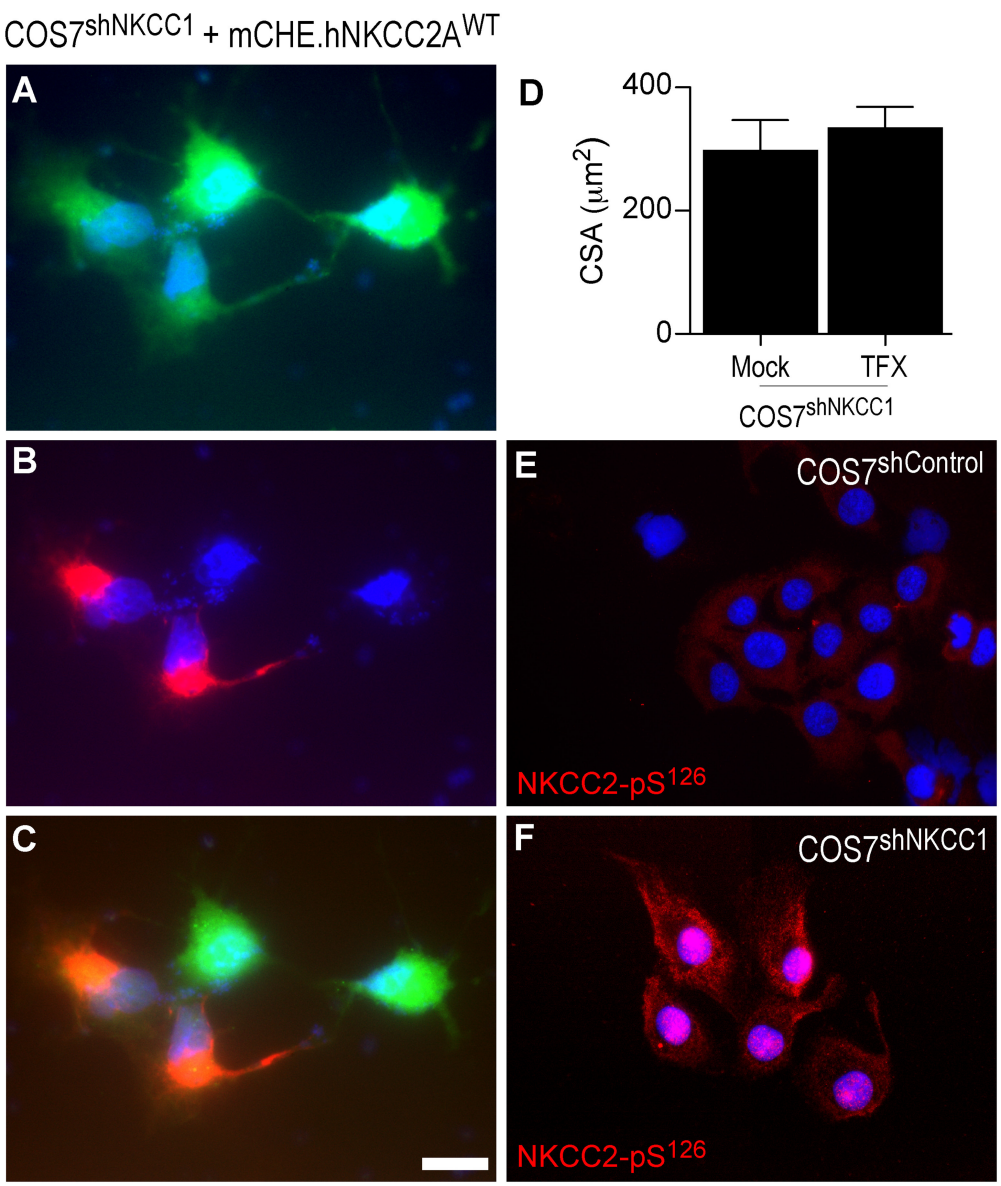
**NKCC2A does not recover the decreased cell size of COS7^***shNKCC***1^ cells. (A–C)** Direct fluorescence microscopy images of COS7^shNKCC1^ cells (p10) grown in glass coverslips and stably expressing GFP from pGIPz-GFP.shNKCC1 constructs **(A)**, transfected with mCherry-hNKCC2A^WT^ **(B)** and superimposed **(C)**. **(D)** Mean CSA in COS7^hNKCC1^ cells transiently transfected with mock or mCherry-hNKCC2A^WT^ (TFX). **(E–F)** Fluorescence microscopy images of COS7^shControl^ **(E)** and COS7^shNKCC1^ **(F)** where endogenous NKCC2 was immunolabeled by using antibodies against the activating phospho-residue pS^126^.

To determine if the decrease in cell size of COS7^shNKCC1^ cells relates to changes in intracellular ionic composition, the basal cellular Cl^−^ content was estimated. Figure 8D shows that BTD decreased total Cl^−^ content of COS7^shControl^ but not in COS7^shNKCC1^ cells, suggesting that NKCC2 may not participate in the maintenance of basal cellular Cl^−^ content in a BTD-sensitive manner, in spite of the fact in these cells NKCC2 was up-regulated (Figure 8A) and localized in the plasma membrane region (Figure 8E) or upon long-lasting pharmacological inhibition of NKCC1 (Alshahrani et al., 2015). Together, these results suggest that silencing of NKCC1, but not NKCC2, impacts cell size and total Cl^−^ content. This is consistent with the notion that NKCC2 and NKCC1 are not functionally equivalent, in terms of cell water volume control (Hamann et al., 2010; Zeuthen and Macaulay, 2012) and BTD sensitivity (Gamba, 2005). To test this hypothesis, shrunken COS7^shNKCC1^ cells were transiently transfected with mCherry-hNKCC2A^WT^ and cross sectional cell area was measured 36 h post-transfection. As shown in Figures 9A–D, over-expression of mCherry-hNKCC2A^WT^ did not significantly change COS7^shNKCC1^ cell size suggesting that NKCC2A does not restore cell volume in shrunken cells lacking NKCC1. To determine if these effects are related to the phosphorylation state of NKCC2, cells grown in coverslips under isotonic conditions were immunolabeled against activating phospho-residue S^126^ in NKCC2. The results show that NKCC2 expressed in COS7^shNKCC1^ (Figure 9F) but not in COS7^shControl^ (Figure 9E) is phosphorylated in S^126^ suggesting that the transporter is functionally active. These results indicate that NKCC2A and NKCC1 are not redundant co-transporters when co-expressed in the same cell and that plasma membrane NKCC1 maintains cell size whereas NKCC2A does not.

## Discussion

The functional and pharmacological properties of NKCC2 have been defined in amphibian heterologous expression systems such as the *Xenopus laevis* oocyte (Gamba, 2005). Yet, relatively little is known about NKCC2 function in mammalian cells models that endogenously express this co-transporter, such as COS7 cells in culture. Further, the mechanisms involved in *Slc12a1* gene expression and its regulation in mammalian cells are not well understood. Previous work showed that COS7 cells express NKCC2 (Alshahrani et al., 2015), a co-transporter that is generally thought to be expressed exclusively in the kidney's TALH. The present report confirms and extends these results by showing that of the three main NKCC2 splice variants (A, B, F) expressed in kidney, COS7 cells express NKCC2A, whereas NKCC2B and NKCC2F are undetectable (Figures 1A–C). This finding prompted us to study the trafficking and regulation of NKCC2A using COS7 cells as a mammalian expression model system.

The NKCC2 protein was found at low but significant levels in COS7 cells when compared to kidney, where the co-transporter is particularly abundant. For instance, to detect NKCC2 in Western blots, 100 μg of protein extracts from COS7 cells were required, instead of 10 μg from kidney (Figure 1D). NKCC2 immunoblots of protein extracts from COS7 cells exhibited a molecular mass band pattern consistent with the transporter being expressed as a protein with various degrees of N-glycosylation (Figure 1D). However, most of NKCC2 from COS7 cells (densitometrically estimated 80–90%) appeared as core/high mannose (~120 kDa) and hybrid-type N-glycans (~135 kDa), whereas the complex N-glycosylated version of the transporter (~160 kDa) was barely detected. These results are consistent with the relatively low expression levels of the transporter in these cells and with the concept that complex N-glycosylation depends on the level of cargo reaching the complex N-glycosylation machinery of the Golgi, as has been suggested for many proteins (Stanley, 2011), including NKCC1 in COS7 cells (Singh et al., 2015) and other ion transporters (Li et al., 2014). The low levels, or even absence, of complex N-glycosylated NKCC2 is consistent with the cellular distribution of the co-transporter in COS7 cells, where most of the endogenous NKCC2 appeared localized in intracellular compartments but not in the plasma membrane. Consistent with the negligible plasma membrane expression is the finding that integrin β2, a plasma membrane marker did not co-localize with NKCC2 (Figures 2A–F), and the co-transporter did not co-localize in lectin-labeled cells (Figures 2G–L). Further, NKCC2 and NKCC1, a co-transporter that localizes to the plasma membrane and in defined intracellular organelles (Maglova et al., 2004; Singh et al., 2015) share some but not all cellular compartments (Figures 2D–F). These results demonstrate that endogenous NKCC2 in COS7 cells is mostly expressed in intracellular compartments.

The above results are consistent with the localization of NKCC2 in intracellular vesicles of the TALH *in vivo* (Nielsen et al., 1998) and in rat medullary TALH cell lines *in vitro* (Eng et al., 2007). In fact, only ~3% of the total TALH NKCC2 localized to the cell surface under basal conditions (Ortiz, 2006). Moreover, endogenous NKCC2 has been found in the perinuclear region of different cell types such as insulin-secreting β-cells of the endocrine pancreas (Alshahrani et al., 2012) and vasopressinergic/oxytocinergic neurons of the hypothalamus (Konopacka et al., 2015). Interestingly, most of the exogenously transfected NKCC2 localizes to the perinuclear region of immortalized cell lines derived from mouse TALH (mKTAL), opossum kidney (OKP) or HEK293 cells (Benziane et al., 2007; Zaarour et al., 2009, 2011). Further, transfected NKCC2 localizes to Rab11-positive vesicles and interacts with secretory carrier-associated membrane protein 2 (Zaarour et al., 2011), which is involved in post-Golgi recycling (van Ijzendoorn, 2006). In addition, it has been shown that NKCC2 interacts with fructose-bisphosphate aldolase B (Benziane et al., 2007), a glycolytic enzyme of demonstrated perinuclear/nuclear location (Sáez and Slebe, 2000). Therefore, these data are consistent with the notion that endogenous NKCC2A is predominantly expressed in intracellular compartments.

In support of the above conclusion, NKCC2A was found in Rab11-positive recycling endosomes (Figures 3A–C) and to a lesser extent in *cis*/*trans*-Golgi cisternae (Figures 3D–I) and the MTOC/centriolar compartment (Figures 3J–L). NKCC2 was not found in lysosomes (Figures 3M–O) or tubulin-positive filamentous structures radiating from the MTOC (Figures 3J–L), a cellular pattern consistent with a protein that follows the “classic” biosynthetic and secretory pathways. NKCC2 was also found in the ER under basal conditions (Figures 4A–C), but minimally after inhibition of global protein synthesis with CHX (Figures 4D–F). However, in contrast with NKCC1 (Singh et al., 2015), inhibition of the first step of N-glycosylation with TUN did not result in retention or degradation of NKCC2 in ER-like structures (Figure 5). In fact, TUN treatment surprisingly redistributed endogenous NKCC2 to the plasma membrane region of COS7 cells (Figures 6B–D). These findings suggest that N-glycosylation of NKCC2A, either high-mannose, hybrid, or complex, is not required for the transporter to reach the plasma membrane. Even collapsing Golgi cisternae into the ER of COS7 cells with BFA did not preclude NKCC2 transit to the plasma membrane region (Figures 6E–G) demonstrating that the transporter can reach that compartment independently of the classic secretory pathway. These results are not a peculiarity of NKCC2; similar observations have been reported for CFTR, the cystic fibrosis conductance regulator (Yoo et al., 2002) and other proteins that traffic directly from ER to plasma membrane via endosomes, through routes independent of Golgi cisternae (Grieve and Rabouille, 2011). Further, recruiting of NKCC2 to the plasma membrane has been recently shown in neurons of the supraoptic and paraventricular nuclei in rats under conditions in which the Golgi apparatus disintegrates (Iijima, 1970) such as chronic hyperosmotic stress (Konopacka et al., 2015).

There are at least two non-mutually exclusive cellular post-ER routes that may be taken by NKCC2 to reach the plasma membrane: The “classic” secretory pathway and/or an unconventional one that does not require Golgi cisternae. Although the functional significance of such alternative pathways is not clear, the election of one over the other appears not related to the expression levels of NKCC2 or to the ability of the transporter to become N-glycosylated. In fact, hNKCC2A^ΔC^ or hNKCC2A^N446/456Q^ mutants that are normally retained in intracellular compartments when over-expressed in mammalian cells (Benziane et al., 2007; Zaarour et al., 2009) or in COS7 (Figures 7A–C), did escape ER retention and translocate to the plasma membrane region upon sustained hyperosmotic stress (Figures 7D–F). Therefore, endogenous natively expressed, core/high-mannose N-glycosylated and ER-located NKCC2A may represent a pool of the transporter, which may become available under certain physiological conditions such as sustained hyperosmotic stress. In contrast to what we have found for NKCC2A, ~50% of non-glycosylatable rat NKCC2F (rNKCC2F^N442/452Q^) reaches the plasma membrane whereas the rNKCC2F activity is reduced by ~80% when over-expressed in *X. Laevis* oocytes (Paredes et al., 2006). Clearly, these data indicate that ~50% of rNKCC2F^N442/452Q^ still reaches the plasma membrane and that rNKCC2F^N442/452Q^ retains ~20% of co-transport activity. It is possible that endogenous NKCC2A, independently of its initial N-glycosylation state can be targeted to the plasma membrane and/or retrieved from recycling endosomes to the Golgi where it may be subjected to complex N-glycosylation and trafficked back to the plasma membrane, as a fully functional transporter. Taken together, the present data suggest that NKCC2 trafficking and targeting can bypass Golgi cisternae to reach the plasma membrane region of COS7 cells subjected to sustained hyperosmotic stress. Notably, NKCC2 expression and plasma membrane localization increase in response to sustained pharmacologic inhibition of NKCC1 with BTD in COS7 and β-cells (Alshahrani et al., 2015) and Konopacka et al. showed similar results in hypothalamic neurons of rats under chronic hyperosmotic stress (Konopacka et al., 2015).

Our experiments suggest that COS7^shNKCC1^ cells are significantly smaller than control cells (Figures 8A–C). This observation needs to be taken within the following contexts: (i) elimination of NKCC1 in COS7 cells took more than 10 passages (Figures 8A,B), which roughly corresponds to ~8–9 weeks; (ii) once NKCC1 was fully silenced, NKCC2 increased to maximal levels of expression, and (iii) changes in the cellular distribution of NKCC2 occurred in response to sustained (>16 h) exposure to slight (~6.7%) hyperosmotic media. Therefore, the decreased cell size of COS7^shNKCC1^ cells occurred regardless of NKCC2A up-regulation (Figure 8A), suggesting that NKCC2A did not recover cell shrinkage resulting from NKCC1 deficiency. This suggests that COS7^shNKCC1^ cells did not exhibit the typical regulatory volume increase (RVI) widely described in the literature when animal cells are exposed to extreme hyperosmotic solutions (50% hyperosmotic or more). Thus, under the experimental conditions described in the present study RVI was absent in cells lacking NKCC1 that up-regulate NKCC2, a co-transporter that does not transport water. In line with the notion that NKCC2A may not participate in cell water recovery is the observation that insulin-secreting β-cells lacking NKCC1, but expressing low levels of NKCC2, are significantly smaller when compared to those expressing both NKCC1 and NKCC2 (Alshahrani and Di Fulvio, 2012).

These results agree with data showing that, in addition to translocating Na^+^, K^+^, and Cl^−^ ions, NKCC1 does co-transport water (Hamann et al., 2010) whereas NKCC2A does not (Zeuthen and Macaulay, 2012). Therefore, up-regulation of endogenous NKCC2 may restore intracellular concentration of ions without restoring cell water volume in cells undergoing slight but sustained shrinkage. In other words, our results suggest that cells lacking NKCC1 exhibit reduced cellular water and ion content per unit of mass. This hypothesis is supported by the present results showing that COS7^shNKCC1^ cells are shrunken (Figures 8B,C), and have reduced *total* Cl^−^ content expressed as nmol per μg of total cellular protein (Figure 8D) regardless increased NKCC2 expression (Figure 8A) and plasma membrane localization (Figure 8E). However, the ratio cell area/total Cl^−^ content in COS7^shNKCC1^, relative to that of COS7^shControl^ cells, is similar. Further, over-expression of hNKCC2A^WT^ in COS7^shNKCC1^ failed to recover normal cell size (Figures 9A–C, compare Figure 9D against Figure 8A) supporting the notion that NKCC2A and NKCC1 are functionally different in terms of water transport properties. It remains to be determined weather in the absence of NKCC1, NKCC2 functions to keep normal [Cl^−^]_i_ and [K^+^]_i_. In fact, COS7^shNKCC1^ cells expressed negligible BTD-sensitive Cl^−^ uptake (Figure 8D) but they still accumulate Cl^−^, suggesting that NKCC2A is resistant to the action of BTD under the experimental conditions tested. This is counterintuitive, given the fact that NKCC2 is the target of the diuretic effect of BTD (Alvarez-Leefmans, 2012). Interestingly, human NKCC2A and rNKCC2F^N442/452Q^ are 3–4 and ~2 times less sensitive, respectively, to BTD when compared to their homologous NKCC2F variants (Paredes et al., 2006; Carota et al., 2010). Although the BTD-sensitivity profile of non-complex N-glycosylated NKCC2A expressed in non-epithelial cells or in cells subjected to chronic dehydration (Konopacka et al., 2015) or sustained osmotic stress remains unknown, it is tempting to speculate that the apparent refractoriness of NKCC2A to BTD may be required to secure ion concentrations within normal ranges and within a context of reduced cell size or water volume brought about sustained hyperosmotic stress like the one observed in chronic systemic dehydration.

Although the detailed molecular mechanisms whereby cells lacking NKCC1 maintain their cell size reduced remain unknown, NKCCs may have the potential to modulate cell size independently of its ion transport capabilities. It is known that NKCC1 (Garzon-Muvdi et al., 2012) and NKCC2 (Carmosino et al., 2012) directly interact with ezrin/radixin/moesin (ERM) proteins, which have been implicated in the regulation of the cell shape, polarity, migration, and growth (Bretscher et al., 2002). However, ERM proteins are activated acutely and transiently in cells exposed to extreme, non-physiological-hyperosmotic conditions e.g., 600 mOsm i.e., 100% hyperosmotic solutions (Rasmussen et al., 2008). These conditions are in sharp contrast with the modestly high (6 to 7%) hyperosmotic solutions used in the present study, which fall within physiological or physio-pathological ranges. Therefore, it remains possible that an interaction NKCC2-ERM may regulate cell size/shape when NKCC1 is silenced. In addition, cells lacking NKCC1 require longer time to duplicate than WT cells (roughly twice the time). It remains to be determined whether this observation is directly related to reduced water permeability in cells lacking NKCC1 but expressing increased NKCC2. The high activation energy required for water transport across COS7 cell membranes (Peckys et al., 2011) is consistent with the idea that water not only permeates via channels (aquaporins) but also through carrier-mediated mechanisms, such as co-transporters. Therefore, it is also plausible that NKCC1 elimination may decrease COS7 cells water permeability.

## Author contributions

All authors participated in the experimental design, data analysis, and interpretation and revised the manuscript for intellectual content. RS carried out the protein expression analyses and immuno-localization experiments. MMA performed the Cl^−^ uptake studies. SK and MD performed RT-PCR experiments, cloning, and immunofluorescence microscopy. MYA and MD produced, maintained, and characterized stable cells. TG and FJA-L created and provided hNKCC2A^WT^, hNKCC2A^ΔC^, and hNKCC2A^N446/456Q^ expression plasmids. MD conceived the studies and wrote the manuscript. FJA-L and MD edited the final versions of the manuscript.

## Funding

This work was partially supported by funding from the Boonshoft School of Medicine, Wright State University, Emerging Science and Seed Grant Programs 229113 to MD and 226191 to FJA-L, as well as NIH-NIDS Grant NS-29227 to FJA-L.

### Conflict of interest statement

The authors declare that the research was conducted in the absence of any commercial or financial relationships that could be construed as a potential conflict of interest.
