# Supplementary material for: Plasma Membrane Targeting of Endogenous NKCC2 in COS7 Cells Bypasses Functional Golgi Cisternae and Complex N-Glycosylation
Source: Front Cell Dev Biol. 2017 Jan 4;4:150. doi: 10.3389/fcell.2016.00150 (PMC5209364; doi:10.3389/fcell.2016.00150)
Supplement: Supplementary file 1 [file Table1.PDF]

Supplementary Table 1: PCR primer sets are named after the target and followed by numbers indicating the predicted length of the RT-PCR product in base pairs (bp). Sense (forward) and antisense (reverse) sequences of these primers are shown. Primers were designed according to the inferred NKCC2A *RefSeq* nucleotide sequence of the chimpanzee (*Pan troglodytes*, ptNKCC2A, NM_001110839).

Primer set	Sense	Antisense
NKCC2-727	ATTGAATCATCTAGAACAAAAGCCAGGAG	GAATGAAGAGCATGACTCCCCAGATGTTC
NKCC2-438	AGCCAGGAGCTCCCTAATGGAA	ACGGCATCCATGGTGTTGTGG
NKCC2-803	GCTCCCTAATGGAAGCACATTAGTGTTTA	TATCGCAGAAGTTGACAACCCAGTAATAGA
NKCC2-401	CAGTGCCCAGTAATACCAATCGC	AGCTGAACTTGGGGTGACTGC
NKCC2A-540	CCTGGGAATCAGGAGTGCTATGA	ACCACCTCCACGAACAAACC
NKCC2-475	ACCATGGATGCCGTTCCCAAG	TCAGGCCTATTGACCCACCGA
NKCC2-612	AGATGATCAAGCTGGTGTTGTG	GGCCCAAAGTTTTCTGCAAA
NKCC2-453	ATGGGTGAAAGGTGTGCTGGT	ATGACTTGGGCCTTTGCCTCC
NKCC2-479	TTCGTGGAGGTGGTGCCTACT	GGCACCAGCAAGAATCCCAGT
NKCC2-423	GGAGGCAAAGGCCCAAGTCAT	AACCCACATGCTGCTGAACCA
NKCC2-603	TTTGCAGAAAACTTTGGGCC	GGTGTTCAGTTCCGCAATAAGA
NKCC2-412	TTTGTGTAGGGGCCTGTGTGG	GCAATGGTGTTCAGTTCCGCA
NKCC2-527	GCTGATGAACAATTTCCAGGTC	AGGAGCCCCAGTTCACATCT
NKCC2-528	TTCTGCAACACTCTCCTCCGC	ACGTGGTCTTCCACTGTGGTT
NKCC2-503	TGCTTCCATGCCTCTTATGCCA	GCCGCCACTGCAGCATAAAAA
NKCC2-870	GCTGTCATCACCTATGTCATTGAATTCTTC	AAACAACCACCAAACATCAATTGTGCCTTT
NKCC2-553	AAGCCAGATGTGAACTGGGGC	CCTCTTGCACCTGAAGAACCTGA
NKCC2-624	AGATGTGAACTGGGGCTCCT	CCACTTTCCTCTTCACACTCAGTATC
NKCC2-526	GGAGATGAACAGTGGCATGGTGA	TTGAGTGCTGGCTTCCACCAG
NKCC2-850	GAGATTGAGAACTACGTGGGAATCATACA	CCAACCAAGCCATATACAACAAATCCGATA
NKCC2-701	TAGTCAGAATCAGCCAAGGATTTGACATC	CTCCTGTAAGAGTTCATTCAGTCGAACTTG
NKCC2-709	GGAAGCAACTATCAAAGATACTGAGTGTG	GAGGATTTCCAACCAAGCCATATACAACAA
NKCC2-452	GGAGGCATCCGAGGCTTGTTT	TTGCAGCTTTCATGGAGACGA
NKCC2-547	AGATGGCAGCATTAACACAAGC	GGGAAGGCTCAGGACAATGA
NKCC2-498	TTGCAGACATCCATATCATCGG	CCAGAGGTTTGCATATCCATAG
NKCC2-759	GCTGGAAAGTCTTTGAAGAGATGATTGAA	CAATAAGCATTTATTGAGATCCTGCTGTGG
NKCC2-815	GAAAGCTGCAAAGATTTAACAACTGCTGA	CTTTGTAGGTATAAGCCTCTGAGGGGG
NKCC2-470	TCCTCACAAAGAACCTCCCACCT	TGCTGTGGGGCTGAGACCTAT
