# Supplementary material for: Plasma Membrane Targeting of Endogenous NKCC2 in COS7 Cells Bypasses Functional Golgi Cisternae and Complex N-Glycosylation
Source: Front Cell Dev Biol. 2017 Jan 4;4:150. doi: 10.3389/fcell.2016.00150 (PMC5209364; doi:10.3389/fcell.2016.00150)

Supplementary Figure 1

Supplementary Figure 1: Nucleotide and deduced amino acid sequence of NKCC2A mRNA expressed in COS7 cells. Shown is the nucleotide sequence of NKCC2A mRNA (3,934 bp) cloned from COS7 cells (GenBank JQ708180). The cDNA contains partial 5' and 3'-UTRs (*nt* 1-112 and 3,413-3,934, respectively) and an open reading frame (ORF) of 3,300 bp (*nt* 113-3412) encoding a predicted 1,099 amino acid protein represented with the single letter code. A poorly conserved Kozak's consensus sequence and a canonical poly-adenylation site are shown as underlined nucleotides located in the 5'- and 3'-UTRs, respectively. Boxed residues represent putative transmembrane domains predicted using five different algorithms (TOPCONS, topcons.cbr.su.se). Analysis includes a potential 13th transmembrane domain predicted by one of the three algorithms.

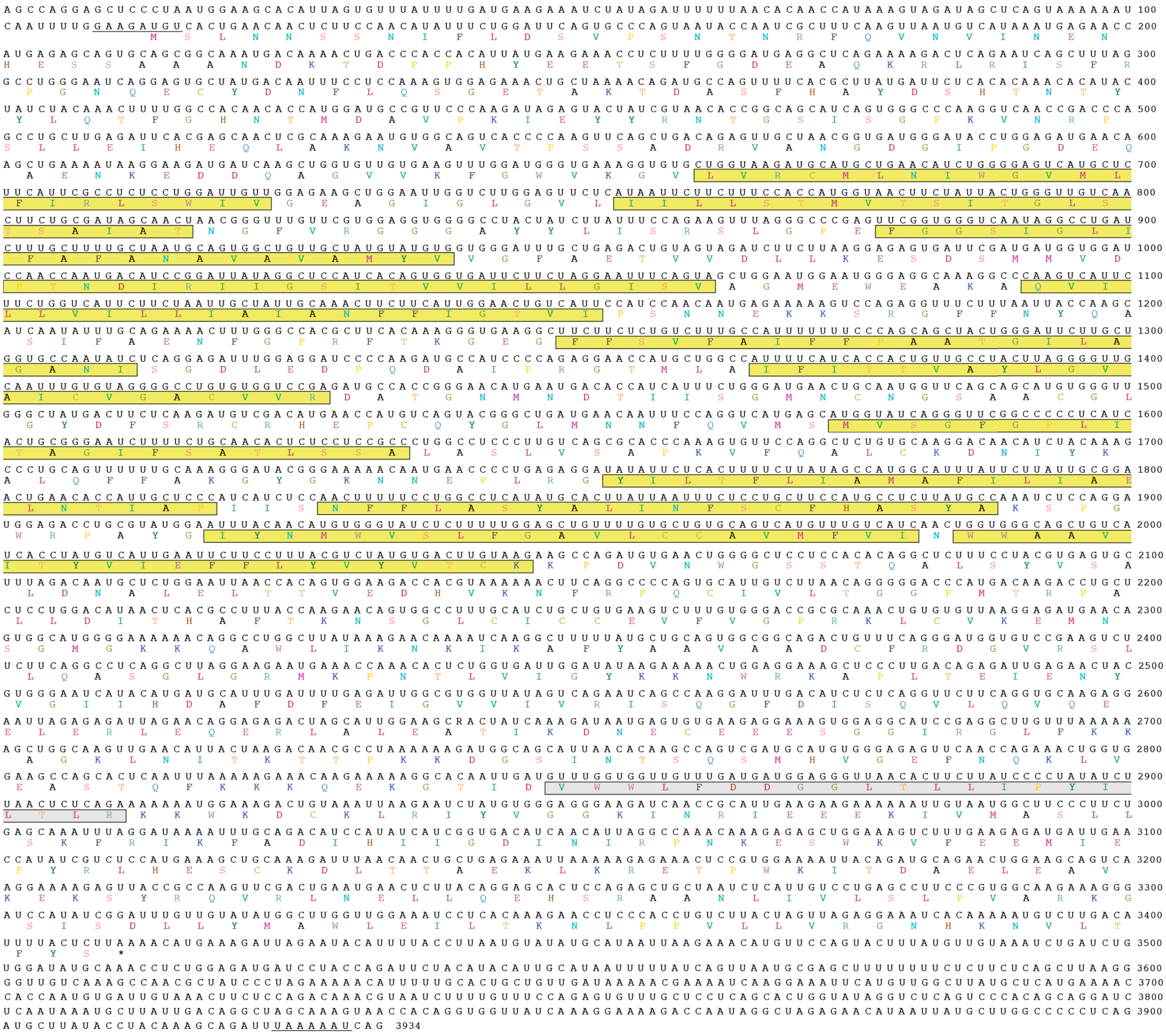

Supplement: Supplementary file 2 [file Image1.PDF]
